# Adult *Zucker* Obese *fa/fa* Rats Present Impaired Immunity and Oxidative-Inflammatory Responses

**DOI:** 10.3390/biom16040547

**Published:** 2026-04-08

**Authors:** Nuria María De Castro, Mónica De la Fuente, Lydia Giménez-Llort, Jaime Ruiz-Tovar, Carmen Vida, María Isabel Baeza

**Affiliations:** 1 Health Sciences Department, San Juan de Dios School of Nursing and Physical Therapy, Comillas Pontifical University, 28036 Madrid, Spain; ibaeza@comillas.edu; 2 San Juan de Dios Foundation, 28036 Madrid, Spain; 3 Department of Genetics, Physiology and Microbiology (Unit of Animal Physiology), Complutense University of Madrid, 28040 Madrid, Spain; mondelaf@bio.ucm.es; 4 Institute of Investigation 12 de Octubre Hospital (Imas12), 28041 Madrid, Spain; 5 Department of Psychiatry and Forensic Medicine, School of Medicine, Autonomous University of Barcelona, 08193 Barcelona, Spain; lidia.gimenez@uab.cat; 6 Institute of Neuroscience (INc), Autonomous University of Barcelona, 08193 Barcelona, Spain; 7 Department of Biomedicine and Health Sciences, Alfonso X University, 28691 Madrid, Spain; jruiztovar@gmail.com; 8 Department of Biology (Animal Physiology Area), Faculty of Sciences, Autonomous University of Madrid, 28049 Madrid, Spain; mariac.vida@uam.es; 9 University Institute of Molecular Biology (IUBM), Autonomous University of Madrid, 28049 Madrid, Spain

**Keywords:** obesity, *Zucker fa/fa* rats, immune dysfunction, inflammation, redox imbalance, premature aging

## Abstract

Background: Obesity involves an excessive buildup of adipose tissue and is linked to chronic inflammation and oxidative stress, both of which contribute to immunosenescence. Obesity and aging share common features, including immune system impairment and oxidative and inflammatory states, suggesting that obesity may represent a model for accelerated immunosenescence. Objectives/Methods: The aim of this research was to evaluate in *Zucker fatty* (*fa/fa*) rats, a well-established genetic model of obesity, multiple immune function parameters (phagocytic activity, natural killer cell function, lymphocyte proliferation in response to mitogens, and cytokine profiles), as well as redox parameters (total antioxidant capacity, glutathione levels, activities of glutathione peroxidase and reductase, and xanthine oxidase activity) in peritoneal leukocytes, spleen, thymus, and liver at adult age (24 weeks). Comparisons were made with *Zucker lean* controls (*fa/+*), commonly used as standard controls, and *Wistar* rats as an independent control group. Results: *Zucker fa/fa* rats displayed significant physiological disorders, including increased body and organ weights, premature immunosenescence characterized by impaired innate and adaptive immune responses, reduced IL-2 and IL-10 secretion, elevated TNF-α production upon mitogen stimulation, and oxidative stress evidenced by redox imbalance in the spleen, thymus, and liver. Conclusions: These immune dysfunctions and oxidative imbalances are comparable to those observed during the aging process. Given that the immune parameters analyzed are considered indicators of health, aging rate, and longevity, our findings suggest that adult *Zucker fa/fa* rats could exhibit features of premature aging.

## 1. Introduction

Obesity, type 2 diabetes and metabolic syndrome are tightly interrelated conditions that exert a major impact on health, especially during aging [1]. Metabolic syndrome is defined by a combination of risk factors such as dyslipidemia, hypertension, and a pro-inflammatory state associated with impaired glucose regulation. Insulin resistance, chronic inflammation, and obesity are the three central elements that converge to drive the metabolic disturbances involved in its onset [1,2,3].

Obesity is recognized as a chronic inflammatory disorder and represents a major risk factor linked to insulin resistance and the development of type 2 diabetes mellitus [4,5]. The prevalence of both conditions has been steadily increasing worldwide [6,7]. Moreover, the metabolic dysfunctions they cause resemble the alterations [8,9] and the oxidative-inflammatory state characteristic of aging [7,10]. Indeed, obesity and type 2 diabetes are considered age-associated diseases [7,11].

Aging is a biological process marked by progressive and generalized decline in organism’s functions, reducing the ability to adapt to changes and maintain homeostasis. While all physiological systems deteriorate with age, those most responsible for homeostatic regulation, the nervous, endocrine, and immune systems are particularly affected. The immune system undergoes age-related changes known as immunosenescence, which influence the rate of aging and modulate the oxidative-inflammatory processes underlying it [12,13]. Chronic inflammatory and oxidative conditions, including obesity, are positively associated with aging, inflammation, reactive oxygen species (ROS), and oxidative stress, ultimately contributing to immunosenescence [7,9,14].

Although aging begins in adulthood, its progression depends on the state of homeostatic systems at that stage [15]. Experimental animals exposed to diet-induced obesity in adulthood display features of accelerated aging [16,17,18,19], supporting the notion that obesity can serve as a model of premature immunosenescence [20].

Animal models are indispensable for advancing the understanding of obesity and type 2 diabetes and their link to premature aging. Rodents, particularly mice and rats, are favored due to their anatomical, physiological, and genetic similarities to humans [21]. These models allow researchers to explore the complex biological mechanisms of obesity and diabetes, including their effects on metabolism, inflammation, oxidative stress, and immune function.

Several monogenic rat models of obesity exist, such as *Koletsky* (*fak/fak*) rats (Obese Spontaneously Hypertensive Rat), *GH-deficient dwarf* (*dw/dw*) rats, *Dahl salt-sensitive* (DS)/obese rats, *Wistar fatty* rats (*fa/fa*) and *Zucker diabetic fatty* (*fa/fa*) rats (*ZDF*) [22,23]. These models provide valuable insights into how obesity contributes to premature aging.

The *Zucker diabetic fatty* (*ZDF*) rat, also known as *Leprfa*, carries a homozygous autosomal mutation in the leptin receptor gene (*fa/fa*) on chromosome 5, resulting in a truncated, non-functional receptor [22,23]. Consequently, *ZDF* rats exhibit hyperphagia, variable hyperglycemia, and glucose intolerance, developing obesity at a juvenile age (3–5 weeks) due to uncontrolled appetite and reduced energy expenditure [22]. Excess fat accumulation and inflammation in white adipose tissue contribute to insulin resistance in this strain, worsening diabetes progression [23]. Thus, *ZDF* rats are a well-established model for studying obesity-related type 2 diabetes [23,24]. As they age, they show organ dysfunction [23,24], including neurodegeneration [25], impaired kidney function, cardiovascular alterations and liver disease [23]. These physiological impairments are accompanied by disrupted metabolic pathways, heightened oxidative stress, and inflammation in organs such as the liver, adipose tissue, brain, heart and kidneys [26,27,28,29,30].

Immune studies in *ZDF* rats mainly focus on macrophages and T cells within adipose tissue [31]. Dysfunctional adipocytes release cytokines such as tumor necrosis factor α (TNF-α), IL-6 and monocyte chemoattractant protein 1 (MCP-1), driving adipose tissue inflammation and promoting cellular senescence [32]. However, immunosenescence in *ZDF* rats has been scarcely investigated in immune organs. Limited studies report abnormal spleen function and altered cytokine profiles [33], along with reduced T cell numbers in blood, spleen, and thymus, and impaired splenocyte proliferation in response to mitogens [34]. A deeper understanding of immune homeostasis in *ZDF* rats is therefore needed to clarify disease progression and complications in obesity and diabetes. Studying immune function from early adulthood can reveal mechanisms underlying premature aging and immunosenescence in these conditions.

The present study aims to characterize a broad extent of immune functions (phagocytic efficiency, natural killer activity, lymphoproliferative responses to mitogens, and cytokine profile) and redox parameters (total antioxidant capacity, glutathione levels, glutathione peroxidase and reductase activities, and xanthine oxidase activity) in peritoneal leukocytes, spleen, thymus, and liver of *Zucker diabetic fatty* (*fa/fa*) rats.

*ZDF* rats were compared with *Zucker lean* controls (*fa/+*), which share the same genetic background but carry the heterozygous leptin receptor mutation (*fa/+*) and do not develop obesity, making them a standard control group [29]. Additionally, since *Wistar* rats have been proposed as potentially more suitable controls for *ZDF* (*fa/fa*) rats than *Zucker lean* (*fa/+*) rats [35], they were included as an independent control in this study.

## 2. Materials and Methods

### 2.1. Animal Procedure

Male *Zucker* obese (*fa/fa*), *Zucker lean* (*fa/+*), and *Wistar* rats (WT) (*n* = 12 per group) were sourced from Harlan Ibérica (Barcelona, Spain) at 5 weeks of age. The animals were housed for 19 weeks in polycarbonate cages under a reversed 12 h light/dark cycle, at a constant temperature of 22 ± 2 °C, until they were sacrificed at adulthood (24 weeks) by decapitation, following the European Community Council Directives (86/609/EEC) on the care and use of laboratory animals for experimental purposes, as well as Spanish animal protection regulations (1201/2005 RD).

All rats had free access to a standard laboratory diet (A04 diet, Panlab L.S., Barcelona, Spain). *Zucker* rats classified as *fa/fa* carry a homozygous recessive mutation in the leptin receptor gene, which leads to obesity, whereas *Zucker lean* rats (*fa/+*) carry the heterozygous mutation and remain non-obese, serving as controls for the *fa/fa* group [22,23,29]. *Wistar* rats were included as an additional independent control model, as they have been suggested to provide a more suitable reference than *Zucker lean* rats [35].

### 2.2. Collection of Peritoneal Leukocytes

Peritoneal cell suspensions were obtained following animal sacrifice. The abdominal area was disinfected with 70% ethanol, and 3 mL of sterile Hank’s solution, pre-warmed to 37 °C and adjusted to pH 7.4, was injected into the peritoneal cavity. After gently massaging the abdomen, the skin and abdominal muscles were opened, and the peritoneal fluid was aspirated using a syringe (approximately 80% of the injected volume was recovered). Leukocytes present in the peritoneal suspension were counted using Neubauer chambers (BLAUBRAND, Wertheim, Germany) under optical microscopy.

The suspensions were adjusted to a final concentration of 5 × 10^5^ macrophages per ml in Hank’s solution or 10^6^ leukocytes per ml in RPMI 1640 complete medium, enriched with L-glutamine (PAA Laboratories, Pasching, Austria) and supplemented with 10% heat-inactivated fetal calf serum (56 °C for 30 min; Gibco, Grand Island, NY, USA) and 1% gentamicin (Gibco, Grand Island, NY, USA).

Macrophages and lymphocytes were identified based on morphological criteria. Cell viability was assessed using the trypan blue exclusion test (Sigma-Aldrich, Madrid, Spain) and was consistently above 98%.

The peritoneal cavity is a multifaceted microenvironment that contains various immune cell populations, including T, B, NK, and various myeloid cells, such as macrophages. In the present study, all immune function assays were performed using unfractionated peritoneal leukocytes, as this approach better preserves the physiological environment surrounding immune cells in vivo [36].

### 2.3. Collection of Tissue Samples and Leukocytes Suspensions

The absolute body weight of each animal was recorded prior to sacrifice, and afterward, the weights of individual organs (white adipose tissue, liver, spleen, thymus) were measured.

Spleen, thymus, and liver were aseptically excised, cleared of fat, and divided into separate tissue samples. For immune function analysis in spleen and thymus, fresh tissue was homogenized in phosphate-buffered saline (PBS). To remove erythrocytes, spleen cell suspensions were centrifuged on a Ficoll-Hypaque gradient (Sigma-Aldrich, Madrid, Spain) with a density of 1.070 g/mL. Leukocytes were collected from the interface, washed, and resuspended in PBS by an additional centrifugation step. The final cell concentration was adjusted to 10^6^ cells/mL and resuspended in RPMI 1640 complete medium supplemented with L-glutamine (PAA Laboratories, Pasching, Austria), 10% heat-inactivated fetal calf serum (56 °C for 30 min; Gibco, Grand Island, NY, USA), and 1% gentamicin (Gibco, Grand Island, NY, USA).

Thymus cell suspensions were processed similarly, although density gradient separation was unnecessary due to the absence of erythrocyte contamination.

Cell viability was assessed using the trypan blue exclusion test (Sigma-Aldrich, Madrid, Spain) and consistently exceeded 98%.

Samples intended for redox state analysis (spleen, thymus, liver) were stored at −80 °C until use. These analyses were performed on tissue homogenates prepared with an electric homogenizer at low speed to minimize heat generation and prevent oxidation. All homogenization steps were conducted in a cold chamber (4 °C), and samples were kept on ice throughout the procedure.

### 2.4. Immune Function Parameters

#### 2.4.1. Phagocytosis Assay

Phagocytic activity toward inert particles (latex beads) was evaluated using a previously described method [37]. Aliquots of 200 μL of peritoneal suspension, adjusted to 5 × 10^5^ macrophages/mL in Hank’s medium, were incubated in migration inhibitory factor (MIF) plates (Kartell, Noviglio, Italy) for 30 min. After incubation, adherent monolayers were washed and resuspended in 200 μL of Hank’s medium plus 20 μL of latex bead solution (Sigma-Aldrich, Madrid, Spain). Following a further 30 min incubation, plates were washed, fixed, and stained. The percentage of macrophages that had ingested at least one latex bead was determined by optical microscopy and expressed as phagocytic efficiency (PE).

#### 2.4.2. Natural Killer Activity Assay

Natural Killer (NK) cell activity was quantified using an enzymatic colorimetric kit (Cytotox 96™ Promega Corporation, Madison, WI, USA), based on lactate dehydrogenase (LDH) activity measured with tetrazolium salts, as previously described [38,39]. Briefly, target cells (YAC-1 murine lymphoma cells) were seeded in 96-well U-bottom plates (Nunclon, Roskilde, Denmark) at 10^5^ cells/well in RPMI 1640 without phenol red (PAA Laboratories, Pasching, Austria). Effector cells (leukocytes from peritoneum, spleen, and thymus) were added at 10^6^ cells/well, achieving an effector-to-target ratio of 10:1. Each sample was tested in triplicate. Plates were centrifuged at 250× *g* for 4 min to promote cell contact and incubated for 4 h. After incubation, plates were centrifuged again at 250× *g* for 4 min, and LDH activity was measured in supernatants by adding the substrate and reading absorbance at 490 nm. Three control conditions were included: target spontaneous release, target maximum release, and effector spontaneous release.

#### 2.4.3. Lymphoproliferation Assay

Lymphocyte proliferation in response to the mitogens Concanavalin A (ConA), lipopolysaccharide (LPS), and Phytohemagglutinin (PHA) was evaluated using a previously described protocol [38]. Aliquots of 200 μL of lymphocyte suspension (10^6^ cells/mL) prepared in complete medium from spleen or thymus were distributed into 96-well flat-bottom microtiter plates (Nunclon, Roskilde, Denmark). Cells were incubated with 20 μL of complete medium (control) or with ConA (1, 3, 5 μg/mL; Sigma-Aldrich, Madrid, Spain), LPS (1, 3, 5 μg/mL; Sigma-Aldrich, Madrid, Spain), or PHA (5, 25, 50 μg/mL; Sigma-Aldrich, Madrid, Spain) for 48 h at 37 °C in a humidified atmosphere containing 5% CO_2_. Subsequently, 0.5 μCi of ^3^H-thymidine (ICN, Costa Mesa, CA, USA) was added to each well, and after 24 h, cells were harvested using an automated harvester (Skatron Instruments, Lier, Norway). Thymidine incorporation was quantified in a beta counter (LKB Instruments, Uppsala, Sweden) for 1 min, and results were expressed as counts per minute (cpm). Each condition was tested in triplicate.

#### 2.4.4. Cytokine in Response to Stimuli

Cytokine concentrations, including the lymphocyte growth factor interleukin-2 (IL-2), the proinflammatory tumor necrosis factor-α (TNF-α) and the anti-inflammatory interleukin-10 (IL-10) were measured in supernatants from lymphocyte cultures under basal conditions and after stimulation with ConA and LPS (1 μg/mL). Following 48 h of incubation with mitogens, supernatants were collected and stored at −20 °C until analysis [38]. Cytokine levels were determined using an immunoassay based on the Luminex^®^ 200™ system and the MILLIPLEX™ MAP commercial kit (Millipore Corporation, Billerica, MA, USA). Results were expressed in pg/mL.

### 2.5. Redox Parameters

#### 2.5.1. Total Antioxidant Capacity Assay

Total antioxidant capacity (TAC) in spleen and liver were determined using a commercial kit (Nanjing Jiancheng Bioengineering Institute, Nanjing, China). Homogenates of spleen and liver were prepared at a concentration of 100 mg/mL in 50 mM phosphate buffer. The assay is based on the reduction in Fe^3+^ to Fe^2+^ by antioxidants present in the sample. The amount of Fe^2+^ formed is quantified by measuring the colored complex generated with phenanthrene, which exhibits an absorbance at 520 nm. The protocol was carried out following the manufacturer’s recommendations and instructions, using a sample volume of 100 μL of tissue supernatant in all assays. Results were expressed as units of total antioxidant capacity per milligram of tissue (U TAC/mg tissue). One unit of Total Antioxidant Capacity is defined as the increase in absorbance of the reaction of 0.01 per minute at 37 °C per milliliter of tissue supernatant.

#### 2.5.2. Total Glutathione Assay

Total intracellular glutathione, the primary non-enzymatic reducing agent in the organism, was quantified using the Tietze enzymatic recycling method [40], with minor modifications [41]. Tissue homogenates were resuspended in a solution containing 5% trichloroacetic acid (TCA, Panreac, Barcelona, Spain) in 0.01 N HCl (degassed with helium for at least 10 min) and adjusted to the following concentrations in spleen (25 mg/mL), thymus (50 mg/mL), and liver (10 mg/mL). Samples were centrifuged at 3200× *g* for 5 min at 4 °C. Aliquots of the supernatant were then analyzed using a reaction mixture composed of 5,5′-dithiobis (2-nitrobenzoic acid) (DTNB, 6 mM; Sigma-Aldrich, Madrid, Spain), β-nicotinamide adenine dinucleotide phosphate, reduced form (β-NADPH, 0.3 mM; Sigma-Aldrich, Madrid, Spain), and glutathione reductase (10 U/mL; Sigma-Aldrich, Madrid, Spain). The reaction was monitored for 240 s, and absorbance was measured spectrophotometrically at 412 nm. Results were expressed as nmol/mg tissue.

#### 2.5.3. Glutathione Peroxidase Activity Assay

Glutathione peroxidase (GPx) activity, a key enzyme in the glutathione cycle that enables its antioxidant function, was determined using the original method by Lawrence and Burk [42], with modifications [39]. This assay is based on the oxidation of GSH by GPx in the presence of cumene hydroperoxide (Sigma-Aldrich, Madrid, Spain). Tissue homogenates were resuspended in 50 mM phosphate buffer (degassed with helium for at least 10 min) and adjusted to the following concentrations in spleen (25 mg/mL) and liver (10 mg/mL). Samples were centrifuged at 3200× *g* for 20 min at 4 °C, and supernatants were used for the assay.

The reaction mixture contained GSH (4 mM; Sigma-Aldrich, Madrid, Spain), glutathione reductase (GR, 1 U/mL; Sigma-Aldrich, Madrid, Spain), β-NADPH (0.2 mM; Sigma-Aldrich, Madrid, Spain), EDTA (1 mM; Sigma-Aldrich, Madrid, Spain), and sodium azide (4 mM; Sigma-Aldrich, Madrid, Spain). The reaction was monitored spectrophotometrically for 300 s by measuring the decrease in absorbance at 340 nm due to NADPH oxidation. Results were expressed as milliunits of enzymatic activity per mg tissue (mU GPx/mg tissue), where one mU of GPx activity is defined as the amount of enzyme that catalyses the oxidation of 1 nmol of NADPH per minute under the assay conditions.

#### 2.5.4. Glutathione Reductase Activity Assay

Glutathione reductase (GR) activity was assessed using the method described [43], with modifications [39]. This assay is based on the reduction in oxidized glutathione (GSSG) by GR and the concomitant oxidation of NADPH.

Tissue homogenates were resuspended in oxygen-free 50 mM phosphate buffer containing 6.3 mM EDTA (Sigma-Aldrich, Madrid, Spain) and adjusted to the following concentrations in spleen (25 mg/mL), thymus (50 mg/mL), and liver (10 mg/mL). Samples were centrifuged at 3200× *g* for 20 min at 4 °C, and supernatants were used for the enzymatic reaction with GSSG as substrate. Total activity was determined by monitoring NADPH oxidation spectrophotometrically at 340 nm. Results were expressed as milliunits of enzymatic activity per mg tissue (mU GR/mg tissue), where one mU of GR activity is defined as the amount of enzyme that catalyses the oxidation of 1 nmol of NADPH per minute under the assay conditions.

#### 2.5.5. Xanthine Oxidase Activity Assay

Xanthine oxidase (XO) activity was measured by fluorescence using the commercial kit “Amplex Red Xanthine/Xanthine Oxidase Assay Kit” (Molecular Probes, Paisley, UK), as previously described [44], in homogenates of spleen, thymus, and liver. In this assay, XO catalyzes the oxidation of purine bases (xanthine/hypoxanthine) to uric acid and superoxide anion. The superoxide generated spontaneously converts to hydrogen peroxide, which reacts stoichiometrically with Amplex Red reagent in the presence of horseradish peroxidase (HRP) to produce resorufin, a red fluorescent compound.

Tissue homogenates were prepared in 50 mM phosphate buffer (pH 7.4) and adjusted to 50 mg/mL. Samples were centrifuged for 30 min at 4 °C, and the resulting supernatants were used for the assay. For each reaction, 50 μL of supernatant was incubated with 50 μL of working solution containing Amplex Red reagent (100 μM), HRP (0.4 U/mL), and xanthine (200 μM). After 30 min of incubation at 37 °C, fluorescence was measured using a microplate reader with excitation at 530 nm and emission at 595 nm. XO provided in the kit served as the standard. Results were expressed as international milliunits (mU) of enzymatic activity per mg of tissue (mU XO/mg tissue), where one mU of XO activity is defined as the amount of enzyme that generates 1 nmol of hydrogen peroxide per minute under the assay conditions.

### 2.6. Data Analysis

Statistical analyses were performed with the SPSS 19 statistical package (SPSS Inc., Chicago, IL, USA). All data are presented as mean ± standard error (SE). Normality of the samples and homogeneity of variances were checked by the Kolmogorov–Smirnov and Levene analyses, respectively. All datasets met the assumption of normality. Statistical comparisons among the three groups (*Wistar*, *Zucker fa/+*, and *Zucker fa/fa*) were performed using one-way analysis of variance (ANOVA). If significant differences were detected, multiple pairwise comparisons were carried out using Tukey’s post hoc test for homogeneous variances or Tamhane’s T2 post hoc test for unequal variances. A *p*-value < 0.05 was considered statistically significant.

## 3. Results

### 3.1. Body and Organs Weights

**Figure 1:**
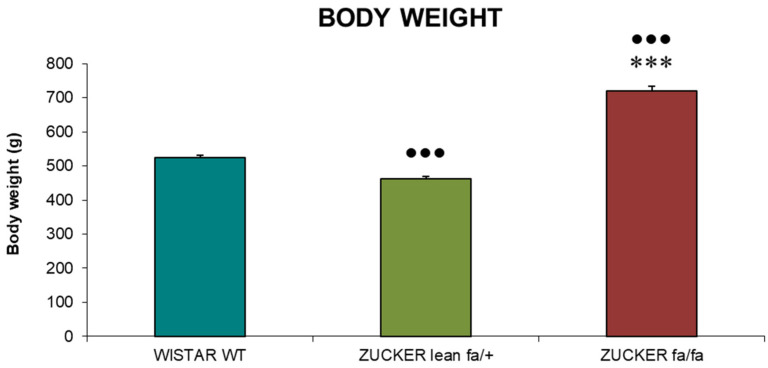
Body weight (g) in *Zucker lean* (*fa/+*), obese (*fa/fa*) and *Wistar* rats (WT) at sacrifice. Data are expressed as mean ± S.E. Statistical analysis was performed by one-way ANOVA followed by Tukey’s post hoc test for homogeneous variances or Tamhane’s T2 post hoc test for unequal variances. *** *p* < 0.001 compared to the corresponding values in *Zucker lean* rats. ●●● *p* < 0.001 compared to the corresponding values in *Wistar* rats.

Body weight values at sacrifice are presented in Figure 1. Obese *Zucker* rats (*fa/fa*) exhibited significantly greater body weights compared with both *Zucker lean* (*fa/+*) controls and *Wistar* (WT) rats (*p* < 0.001). In contrast, *Zucker lean* rats displayed markedly lower body weights than *Wistar* rats (*p* < 0.001).

Table 1 summarizes the measurements of organs collected at sacrifice (white adipose tissue, liver, spleen, and thymus) from lean and obese *Zucker* rats (*fa/fa*) as well as *Wistar* rats. The data include both the absolute organ weights (g) and the relative percentage of each organ weight in relation to total body weight, calculated as: (weight of the organ (g)/body weight of the animal (g)) ×100.

Analysis of absolute organ weights revealed that obese *Zucker* rats (*fa/fa*) had significantly higher values than *Zucker lean* and *Wistar* controls, with the exception of the spleen, which was only heavier relative to *Zucker lean* rats. In these animals, the absolute weights of all organs examined were consistently lower than those of *Wistar* rats.

With respect to relative weights, *fa/fa* rats demonstrated a significantly reduced spleen-to-body weight ratio compared with both *Zucker lean* and *Wistar* rats. Conversely, relative weights of white adipose tissue, liver, and thymus were elevated in *fa/fa* rats compared with both control groups. *Zucker lean* rats exhibited reduced relative weights of white adipose tissue and spleen compared with *Wistar* rats.

### 3.2. Immune Function Parameters

#### 3.2.1. Phagocytic Efficacy and Natural Killer Activity

**Figure 2:**
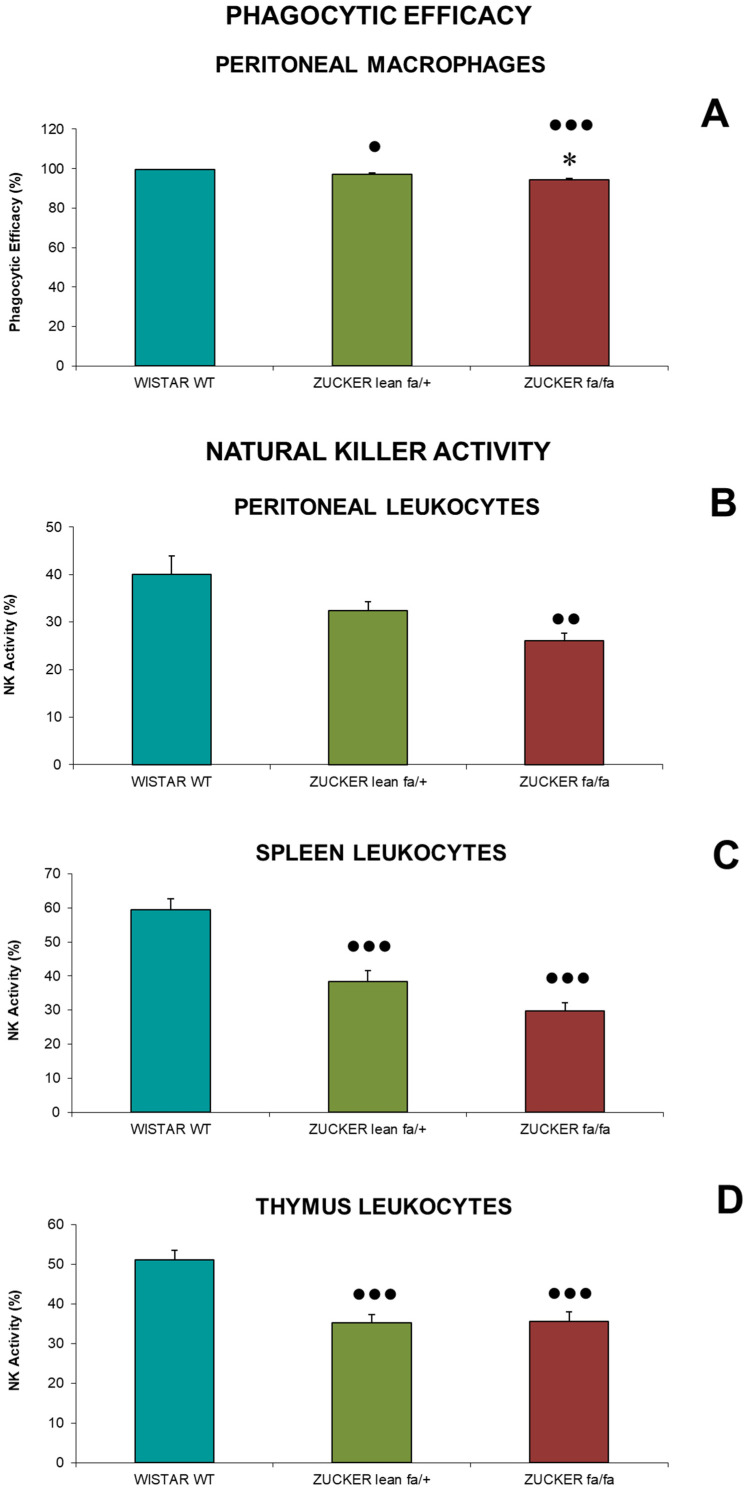
Phagocytic Efficacy (P.E.) of peritoneal macrophages (**A**) and Natural Killer Activity (% lysis) of peritoneal (**B**), spleen (**C**) and thymus (**D**) leukocytes in *Zucker lean* (*fa/+*), obese (*fa/fa*) and *Wistar* (WT) rats. Data are expressed as mean ± S.E. Statistical analysis was performed by one-way ANOVA followed by Tukey’s post hoc test for homogeneous variances or Tamhane’s T2 post hoc test for unequal variances. * *p* < 0.05 compared to the corresponding values in *Zucker lean* rats. ●●● *p* < 0.001, ●● *p* < 0.01, ● *p* < 0.05 compared to the corresponding values in *Wistar* rats.

Phagocytic efficacy (P.E.) of peritoneal macrophages (A) and Natural Killer (NK) cell activity, expressed as the percentage of tumor cell lysis by leukocytes from the peritoneum (B), spleen (C), and thymus (D), are presented in Figure 2.

For phagocytic efficacy (Figure 2A), obese *Zucker* rats (*fa/fa*) showed significantly reduced values (*p* < 0.05) compared to *Zucker lean* controls. Furthermore, both *Zucker lean* rats (*p* < 0.05) and *fa/fa* rats (*p* < 0.001) had lower values than *Wistar* rats, which displayed the highest P.E. value.

Regarding NK activity in peritoneal leukocytes (Figure 2B), *fa/fa* rats exhibited lower activity than *Zucker lean* controls. Additionally, NK activity in *fa/fa* rats was significantly reduced (*p* < 0.01) compared to *Wistar* rats.

In the spleen (Figure 2C), obese *Zucker* rats (*fa/fa*) showed a non-significant reduction in lysis percentage compared to *Zucker lean* rats. However, both *Zucker* groups had significantly lower lysis values than *Wistar* rats (*p* < 0.001). In the thymus (Figure 2D), no differences were observed between obese and *Zucker lean* rats, but both groups demonstrated significant reduced NK activity (*p* < 0.001) compared to *Wistar* rats.

#### 3.2.2. Lymphoproliferation

**Figure 3:**
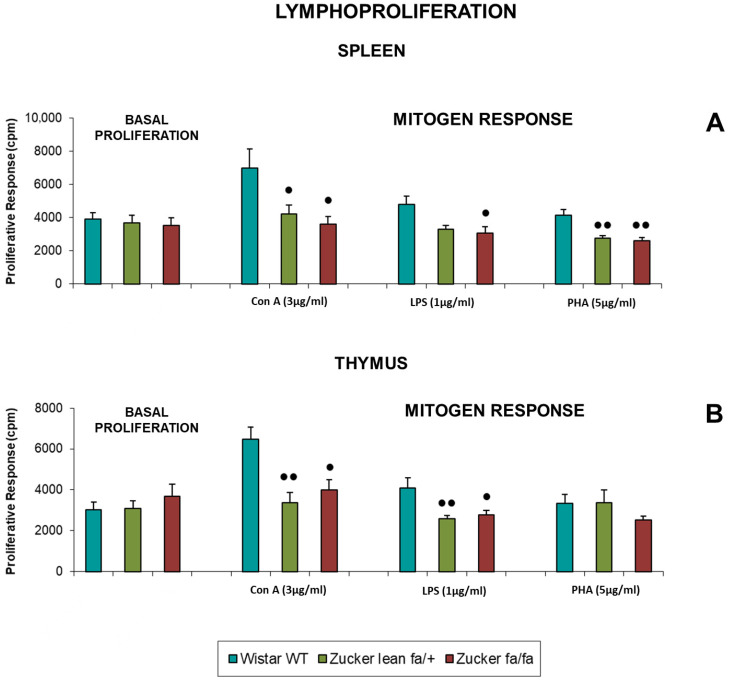
Basal proliferation and mitogen-induced responses (cpm) to Con A (3 μg/mL), LPS (1 μg/mL), and PHA (5 μg/mL) in spleen (**A**) and thymus (**B**) lymphocytes from *Zucker lean* (*fa/+*), obese (*fa/fa*) and *Wistar* (WT) rats. Data are expressed as mean ± S.E. Statistical analysis was performed by one-way ANOVA followed by Tukey’s post hoc test for homogeneous variances or Tamhane’s T2 post hoc test for unequal variances. ●● *p* < 0.01, ● *p* < 0.05 compared to the corresponding values in *Wistar* rats.

Table 2 presents the basal proliferation and mitogen-induced proliferative responses of spleen and thymus lymphocytes to Con A (1 and 5 μg/mL), LPS (3 and 5 μg/mL), and PHA (25 and 50 μg/mL). Figure 3 shows the responses to Con A (3 μg/mL), LPS (1 μg/mL), and PHA (5 μg/mL). All values are expressed in counts per minute (cpm).

In the spleen, obese *fa/fa* rats exhibited lower proliferation than *Wistar* rats, with significant differences upon stimulation with Con A (1, 3, and 5 μg/mL) (*p* < 0.05), LPS (1 μg/mL) (*p* < 0.05), and PHA (5 μg/mL) (*p* < 0.01). No differences were observed between obese and lean rats.

*Zucker lean* rats also showed reduced proliferative responses compared to *Wistar* rats, with significant differences for Con A (3 and 5 μg/mL) (*p* < 0.05) and PHA (5 μg/mL) (*p* < 0.01). Basal proliferation did not differ among groups.

In the thymus, basal proliferation was similar across groups. Upon mitogen stimulation, *fa/fa* rats did not differ significantly from *Zucker lean* rats. However, both *Zucker* groups showed lower proliferative responses than *Wistar* rats, with significant reductions in lean rats (*p* < 0.01) and obese rats (*p* < 0.05) after stimulation with Con A (3 and 5 μg/mL) (*p* < 0.05) and LPS (1 μg/mL).

#### 3.2.3. Cytokines Concentrations

**Figure 4:**
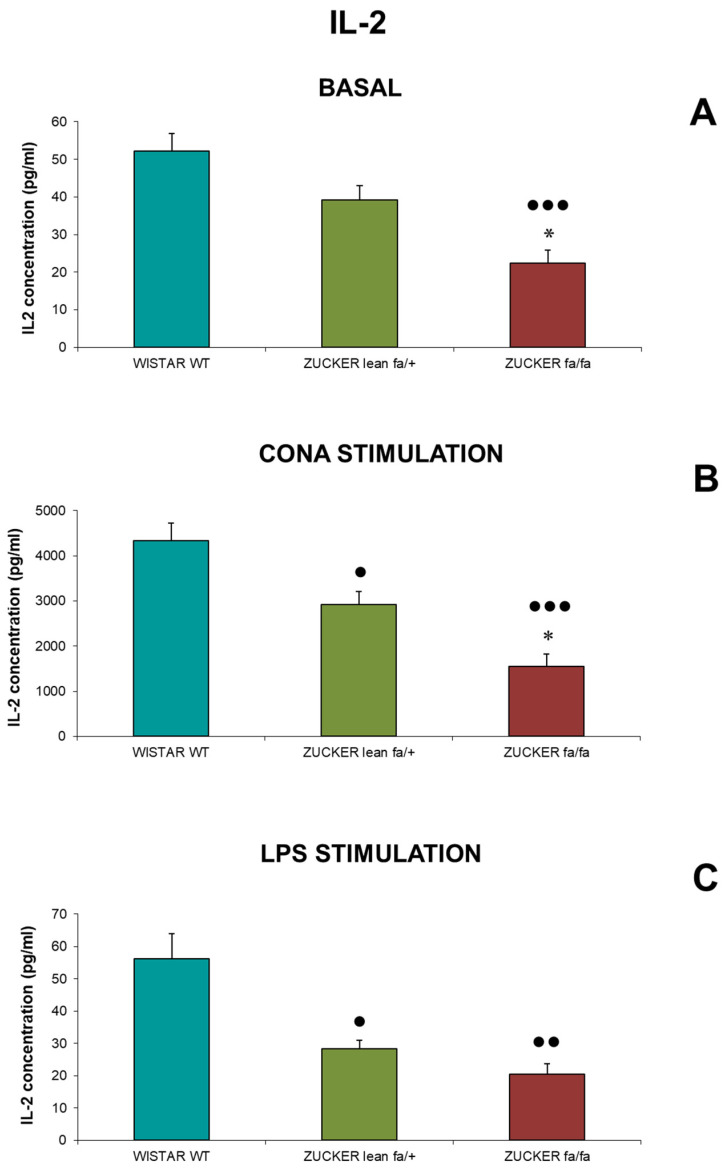
IL-2 concentrations (pg/mL) from spleen lymphocytes under basal conditions (**A**), after Con A stimulation (**B**), and after LPS stimulation (**C**) in *Zucker lean* (*fa/+*), obese (*fa/fa*) and *Wistar* (WT) rats. Data are expressed as mean ± S.E. Statistical analysis was performed by one-way ANOVA followed by Tukey’s post hoc test for homogeneous variances or Tamhane’s T2 post hoc test for unequal variances. * *p* < 0.05 compared to the corresponding values in *Zucker lean* rats. ●●● *p* < 0.001, ●● *p* < 0.01, ● *p* < 0.05 compared to the corresponding values in *Wistar* rats.

IL-2 concentrations (pg/mL) released from spleen lymphocytes are shown in Figure 4. Under basal conditions (Figure 4A), *fa/fa* rats exhibited significantly lower IL-2 concentrations than lean controls (*p* < 0.05). Both *Zucker* groups had reduced concentrations compared with *Wistar* rats, with *fa/fa* rats showing highly significant reductions (*p* < 0.001). Following Con A stimulation (Figure 4B), *fa/fa* rats again released less IL-2 than lean controls (*p* < 0.05), and both *Zucker* groups had lower levels than *Wistar* rats (*p* < 0.05 for lean, *p* < 0.001 for *fa/fa*). LPS stimulation (Figure 4C) produced similar results, with both *Zucker* groups showing reduced IL-2 compared with *Wistar* rats (*p* < 0.05 for lean, *p* < 0.01 for *fa/fa*).

**Figure 5:**
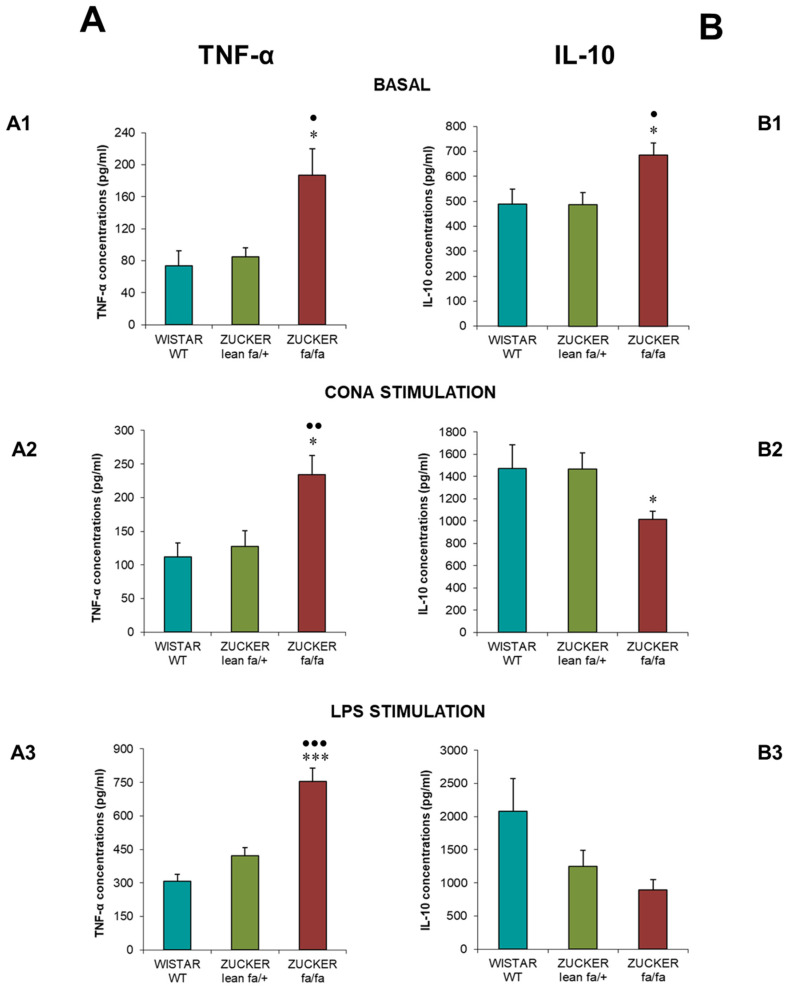
TNF-α (**A**) and IL-10 (**B**) concentrations (pg/mL) from spleen lymphocytes under basal conditions (**A1**,**B1**), after Con A stimulation (**A2**,**B2**), and after LPS stimulation (**A3**,**B3**) in *Zucker lean* (*fa/+*), obese (*fa/fa*) and *Wistar* (WT) rats. Data are expressed as mean ± S.E. Statistical analysis was performed by one-way ANOVA followed by Tukey’s post hoc test for homogeneous variances or Tamhane’s T2 post hoc test for unequal variances. *** *p* < 0.001, * *p* < 0.05 compared to the corresponding values in *Zucker lean* rats. ●●● *p* < 0.001, ●● *p* < 0.01, ● *p* < 0.05 compared to the corresponding values in *Wistar* rats.

Figure 5 represents TNF-α (Figure 5A) and IL-10 (Figure 5B) concentrations (pg/mL). Obese *fa/fa* rats exhibited significantly elevated TNF-α under basal conditions (Figure 5(A1)) compared with lean controls and *Wistar* rats (*p* < 0.05). Following Con A stimulation (Figure 5(A2)), TNF-α remained higher in *fa/fa* rats with respect to lean (*p* < 0.05) and *Wistar* rats (*p* < 0.01). LPS stimulation (Figure 5(A3)) further increased TNF-α in *fa/fa* rats (*p* < 0.001) compared to lean and *Wistar* rats.

Regarding IL-10, *fa/fa* rats released significantly higher basal concentrations (Figure 5(B1)) than lean controls and *Wistar* rats (*p* < 0.05). After Con A stimulation (Figure 5(B2)), however, *fa/fa* rats exhibited lower IL-10 concentrations than lean controls (*p* < 0.05). No significant differences among groups were observed following LPS stimulation (Figure 5(B3)).

### 3.3. Redox Parameters

#### 3.3.1. Spleen Redox State

Redox parameters in spleen homogenates are presented in Table 3. Total antioxidant capacity was significantly reduced in both *Zucker lean* rats (*p* < 0.05) and obese *Zucker* rats (*fa/fa*) (*p* < 0.001) compared with *Wistar* rats. No differences were observed among groups in total glutathione concentrations.

Enzymatic activity analysis revealed that glutathione peroxidase activity was elevated in both *Zucker* groups relative to *Wistar* rats, reaching statistical significance in *fa/fa* rats (*p* < 0.05). Glutathione reductase activity did not differ among groups. Xanthine oxidase activity was significantly increased in *fa/fa* rats compared with lean controls (*p* < 0.01), whereas *Zucker lean* rats exhibited significantly lower activity than *Wistar* rats (*p* < 0.01).

#### 3.3.2. Thymus Redox State

**Figure 6:**
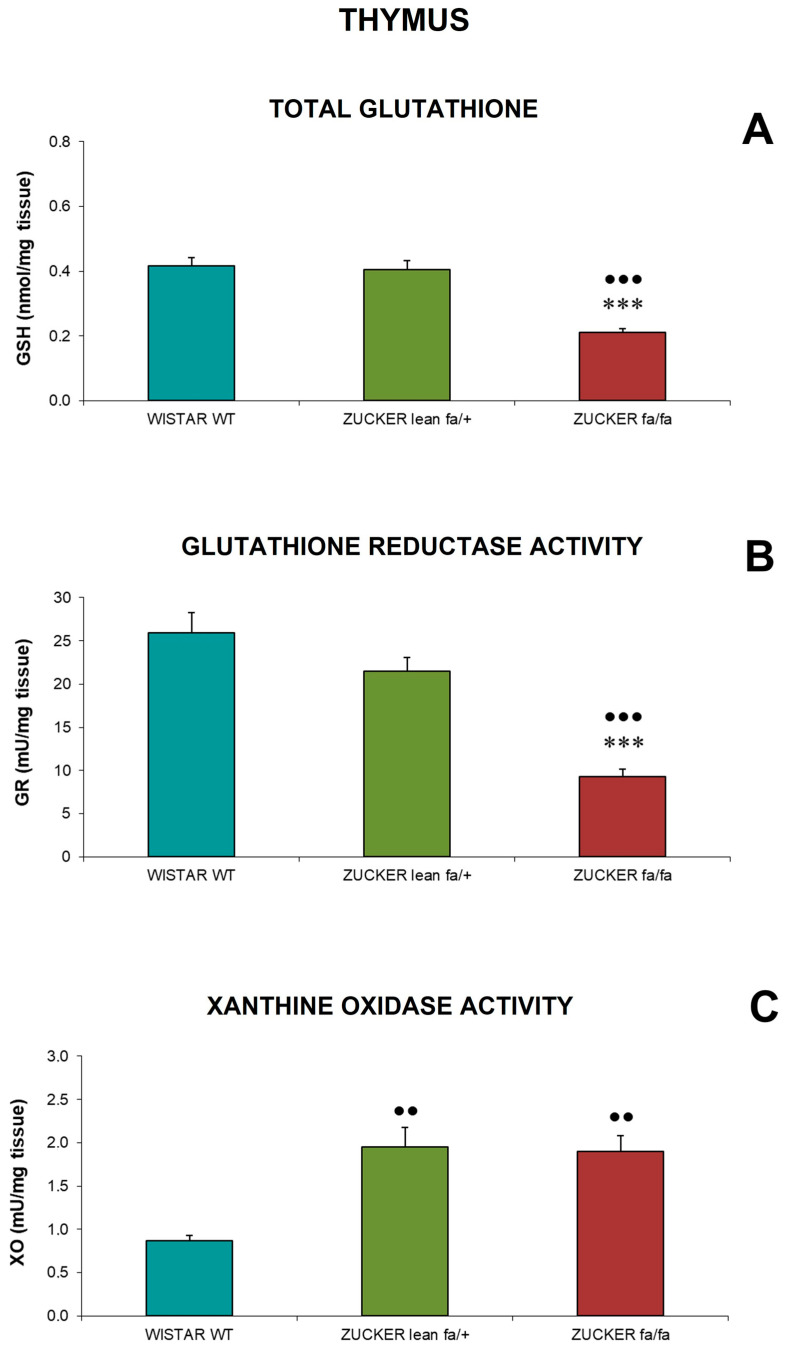
Total glutathione (nmol/mg tissue) (**A**), glutathione reductase activity (mU/mg tissue) (**B**), and xanthine oxidase activity (mU/mg tissue) (**C**) in thymus homogenates of *Zucker lean* (*fa/+*), obese (*fa/fa*) and *Wistar* (WT) rats. Data are expressed as mean ± S.E. Statistical analysis was performed by one-way ANOVA followed by Tukey’s post hoc test for homogeneous variances or Tamhane’s T2 post hoc test for unequal variances. *** *p* < 0.001 compared to corresponding values in *Zucker lean* rats. ●●● *p* < 0.001, ●● *p* < 0.01 compared to corresponding values in *Wistar* rats.

Oxidative stress parameters in thymus homogenates are shown in Figure 6. Total glutathione concentrations (Figure 6A) and glutathione reductase activity (Figure 6B) were markedly reduced in *fa/fa* rats compared with both *Zucker lean* and *Wistar* rats (*p* < 0.001).

Xanthine oxidase activity (Figure 6C) was significantly elevated in both *Zucker* groups relative to *Wistar* rats (*p* < 0.01).

#### 3.3.3. Liver Redox State

Redox parameters in liver homogenates are summarized in Table 4. Total antioxidant capacity did not differ among groups. Total glutathione levels were significantly lower in *fa/fa* rats compared with lean controls (*p* < 0.05).

Glutathione peroxidase activity was significantly reduced in *fa/fa* rats compared with both *Zucker lean* and *Wistar* rats (*p* < 0.01). Similarly, glutathione reductase activity was decreased in *fa/fa* rats relative to lean controls (*p* < 0.05) and *Wistar* rats (*p* < 0.01). No differences were observed between *Zucker lean* and *Wistar* rats for either enzyme.

Xanthine oxidase activity was lower in both *Zucker* groups compared with *Wistar* rats, reaching statistical significance in lean rats (*p* < 0.05).

## 4. Discussion

A joint assessment of multiple immunological functions together with oxidative stress markers was performed in this study, offering an integrated perspective using the *Zucker fa/fa* rat as a model of obesity. Comparisons were made with *Zucker lean* rats (same strain controls) and *Wistar* rats.

### 4.1. Body and Organ Weights

With respect to body weight, previous research indicates that *Zucker fa/fa* rats develop marked obesity as early as 3–5 weeks of age, and by 14 weeks, more than 40% of their body mass consists of lipids when compared with their *Zucker lean* (*fa/+*) littermates. This reflects an early onset of hyperphagia and weight gain [25]. In the present work, body weight was measured in adult rats at 6 months of age (24 weeks), revealing that obese *Zucker fa/fa* rats were significantly heavier than both their lean counterparts and the *Wistar* strain. This observation aligns with other studies reporting increased body weight in this model at the beginning of adulthood (around 20 weeks), consistently showing higher values in obese *Zucker fa/fa* rats relative to lean controls [24,25,30,45,46,47].

Obesity represents a metabolic condition characterized by the expansion of adipose tissue through both adipocyte hyperplasia and hypertrophy [48]. The obese rats displayed greater absolute white adipose tissue mass, as well as a higher percentage relative to total body weight. These animals exhibit hyperplasia and hypertrophy of adipocytes, with the most pronounced rise in cell number occurring in subcutaneous adipose tissue. The findings in this tissue confirm that by 24 weeks, obesity is fully established in this model, and the rise in body weight is largely attributable to subcutaneous adipose tissue accumulation [49].

Recent evidence suggests that adipose tissue contributes to organism aging and age-related diseases [50]. Moreover, it appears to play a key role in obesity-associated immunosenescence [9]. Obesity is positively linked to aging, inflammation, reactive oxygen species (ROS), and oxidative stress, all of which promote immunosenescence [9,11,14]. These findings support the concept that obesity may function as a model of premature immunosenescence [20].

*Zucker fa/fa* rats also exhibited significantly increased liver weight, both in absolute and relative terms, compared with lean controls and *Wistar* rats. Marschall et al. similarly reported higher liver weight in *Zucker fa/fa* rats than in lean animals [47]. This model develops metabolic syndrome and, in addition to obesity, diabetes, and insulin resistance, also presents non-alcoholic fatty liver disease (NAFLD) and hepatomegaly, which account for the increased liver weight [51]. The underlying mechanism involves ectopic lipid accumulation in the liver due to dysfunctional adipose tissue that can no longer store lipids effectively [52]. NAFLD has also been associated with a proinflammatory state in both humans and animal models.

Because immunosenescence in these animals has been studied mainly in macrophages and T cells from adipose tissue rather than in primary immune organs, it was considered relevant to examine spleen and thymus weights. As observed for adipose tissue, obese *fa/fa* rats showed higher absolute spleen and thymus weights than lean controls and *Wistar* rats, indicating hypertrophy. Central obesity and several body composition indicators related to obesity have been associated with increased spleen volume [53]. Moreover, a mouse model of obesity also demonstrated a relationship between obesity and splenomegaly [54]. Splenomegaly has been linked not only to altered immune responses but also to portal hypertension secondary to liver disease.

Regarding relative organ weight, obese rats showed an increased thymus percentage compared with lean and *Wistar* rats but a decreased spleen percentage. This result is consistent with the findings of Ruth et al. [33], who reported that *Zucker fa/fa* rats had higher body and absolute spleen weight than lean controls, but lower spleen weight per gram of body weight. Tanaka et al. [34] documented lymphopenia in peripheral blood, spleen and thymus in *Zucker fa/fa* rats after 8 weeks of age and with advancing age. This may reflect impaired function of thymic stromal cells responsible for T-cell development and maturation, potentially leading to compensatory thymic hypertrophy.

The increased absolute organ weights observed in obese rats suggest that this hypertrophy may be linked to metabolic syndrome. The reduction in relative spleen weight may result from disproportionate organ growth compared with overall body mass expansion, likely influenced by fat accumulation, inflammation, and metabolic dysregulation. In contrast, the marked increases in relative white adipose tissue and liver weight reinforce their central role in obesity-related pathology.

### 4.2. Immune Function Parameters

Analysis of peritoneal leukocyte functions revealed that obese rats displayed a marked reduction in macrophage phagocytic efficiency compared with their lean counterparts and the *Wistar* strain. This indicates that adult obese *fa/fa* rats experience a decline in this component of innate immunity. These findings align with previous research in diet induced obese mice, where peritoneal macrophages also showed impaired phagocytic capacity and reduced Natural Killer (NK) cell activity [16]. NK cell cytotoxicity in peritoneal, splenic, and thymic leukocytes against tumor cells was likewise diminished in *Zucker fa/fa* rats relative to *Wistar* controls. Although *Zucker fa/fa* rats also exhibited slightly lower innate immune function than their lean counterparts, the differences became substantially more pronounced when compared with *Wistar* rats. A similar pattern has been reported in humans with obesity, where NK cell numbers and activity were significantly reduced compared with healthy controls [55]. Such impairments heighten vulnerability to infections and increase the likelihood of obesity associated cancers [56,57,58].

Regarding lymphocyte proliferation in spleen and thymus, this study is the first to employ multiple mitogens at varying concentrations. Although responses were heterogeneous, obese *fa/fa* rats consistently showed reduced proliferation compared with *Wistar* rats when stimulated with different concentrations of Con A and PHA (T cell mitogens) and LPS (B cell mitogen). Despite both Con A and PHA targeting T lymphocytes, *Zucker* rats exhibited a pronounced reduction in Con A induced proliferation, whereas PHA responses did not differ significantly. This discrepancy in responses likely reflects their distinct mechanisms of action. Con A functions as a mitogen that predominantly activates T lymphocytes, showing a more targeted and potent effect in specific T cell subsets. Its activity relies on binding to particular receptors on the T cell surface, triggering their activation and subsequent proliferation. The proliferative response elicited by Con A is typically rapid and robust, especially when T cells are already primed or exposed to additional immunological signals. In contrast, PHA interacts with glycoprotein receptors on T lymphocytes, driving their activation and expansion. The proliferative response induced by PHA tends to be broader and sometimes more sustained, and it may also stimulate other immune cell populations to a lesser degree [59,60].

Other studies have similarly reported impaired splenocyte proliferation in obese *Zucker* rats following stimulation with various mitogens [34,61], as well as reduced responses to PHA and LPS in other rat obesity models [62,63]. Peritoneal leukocytes from diet induced obese mice also showed diminished proliferation in response to Con A and LPS, with basal proliferation remaining unchanged [16], consistent with the present findings in splenic and thymic lymphocytes of obese rats. Human studies likewise demonstrate suppressed T and B cell proliferation in individuals with obesity [64,65]. Altogether, the reduced proliferative capacity observed in obese *fa/fa* rats supports the idea that chronic inflammation associated with obesity dampens adaptive immune responses.

Cytokine secretion by splenic lymphocytes further reflected immune dysfunction. *Zucker fa/fa* rats exhibited significantly reduced basal IL-2 (growth factor) release and diminished IL-2 production following Con A stimulation compared with lean and *Wistar* controls. LPS-induced cytokine release was also impaired, reaching significance only relative to *Wistar* rats. In contrast, TNF-α (proinflammatory cytokine) levels were markedly elevated in obese rats under both basal and stimulated conditions. IL-10, an anti-inflammatory cytokine, was increased in obese rats under basal conditions but decreased following mitogen stimulation, with the reduction reaching significance compared with lean rats after Con A exposure.

These findings are consistent with another work in *Zucker fa/fa* rats showing reduced IL-2 release by splenocytes after Con A stimulation, along with increased TNF-α production in response to Con A and LPS with respect to lean controls. That study also reported elevated IL-1β and IL-6 levels in obese rats, with no differences in IL-10 [33]. In a diet induced rat obesity model, splenic IL-10 synthesis was reduced, likely due to oxidative stress and apoptosis impairing cellular function [66]. This supports the decreased IL-10 response to mitogens observed in obese *fa/fa* rats, although the elevated basal IL-10 levels in the present study may reflect a compensatory response to chronic inflammation. In any case, the inability of splenocytes from obese rats to produce IL-10 upon stimulation indicates clear cellular dysfunction.

A study carried out in an adolescent obese mouse model fed a high fat diet reported reduced IL-2 and IL-10 release from peritoneal leukocytes in adulthood, consistent with the present findings, although TNF-α levels decreased in that model, highlighting variability across obesity animal models [16].

Overall, *Zucker fa/fa* rats displayed a cytokine imbalance shifted toward inflammatory state. Reduced IL-2 production under basal and stimulated conditions suggests impaired T cell activation. Elevated TNF-α, particularly after LPS stimulation, reflects heightened proinflammatory signalling, characteristic of obesity-related inflammation. These findings are consistent with evidence from obese humans, where B cell alterations in composition, differentiation, and function promote immune dysregulation and obesity-related inflammation at both systemic and adipose-tissue levels [67]. They also support evidence that CD4+ and CD8+ T cells play central roles in obesity associated inflammatory processes in both mice and humans [68]. Furthermore, the reduced IL-10 response in obese animals after stimulation further indicates impaired regulatory mechanisms, exacerbating inflammatory damage.

This pattern of cytokine secretion by splenocytes aligns with that observed in expanding adipose tissue in obesity, where adipocytes and infiltrating immune cells-especially macrophages, but also T and B cells-produce increased amounts of proinflammatory adipokines such as TNF-α, IL-6, and IL-1β. These mediators contribute to chronic low-grade inflammation and promote the onset and advancement of metabolic disturbances including insulin resistance [52,69,70,71].

These findings align with extensive evidence showing that metabolic disturbances in obesity share strong similarities with those occurring during aging [32,72]. Both conditions are characterized by chronic systemic inflammation and elevated pro-senescence cytokines (TNF-α, IL-6, C-reactive protein), creating a more oxidative cellular environment [72,73] and reinforcing the close link between inflammation and oxidative processes [74]. This ultimately contributes to the well described chronic “oxi-inflamm-aging” state, marked by persistent and unresolved production of proinflammatory mediators, that increases susceptibility to age related diseases and mortality [73,75].

### 4.3. Redox State Parameters

In the context of obesity and its impact on immune function, it has been proposed that obesity may act as a model of premature immunosenescence [20]. Obesity is characterized by chronic inflammation and oxidative stress, conditions in which the immune system plays a central role due to its production of inflammatory mediators and reactive oxidant molecules during immune defense [12]. This is consistent with the impaired immune responses and altered inflammatory and oxidative profiles described in various diet induced obesity models [18,19,76].

In the present study, obese *Zucker fa/fa* rats displayed several alterations in redox balance. In the spleen, they exhibited reduced total antioxidant capacity together with increased activity of the oxidant enzyme xanthine oxidase. In the thymus, total glutathione levels and glutathione reductase activity were decreased, while xanthine oxidase activity was elevated. Comparable disturbances were detected in the liver, where glutathione peroxidase and glutathione reductase activities were reduced, and glutathione concentrations were lower than in lean controls.

Although the pattern of results was heterogeneous, the overall profile indicates an imbalance in redox state with a shift toward oxidative stress in obese rats. Previous studies in adult *fa/fa* rats have reported disruptions in oxidative inflammatory metabolic pathways in adipose tissue, liver, and heart, three organs central to obesity-related metabolic and vascular dysfunction [26]. These alterations included increased total glutathione in the liver, a trend also observed in the present study, where values did not differ significantly from those of *Wistar* rats, and particularly in the spleen. Given that glutathione is a major antioxidant involved in neutralizing cellular oxidants, regulating immune responses, and supporting DNA synthesis [77,78,79], its elevation may represent a compensatory response to oxidative stress arising from metabolic dysfunction.

Evidence combining oxidative markers and antioxidant enzyme activity in *Zucker fa/fa* rats remains limited. However, a recent study reported heterogeneous redox profiles and variability in the reduced to oxidized glutathione ratio (GSH/GSSG) in plasma and cardiac tissue of aged obese rats, indicating increased oxidative stress [35]. Further research in 38–39-week-old *fa/fa* rats found elevated lipid peroxidation in the kidneys and a tendency toward increased GSH/GSSG ratio in the renal medulla compared with *Wistar* rats [29], consistent with our findings in the spleen. Increased oxidative stress markers have also been documented in the brains of obese rats [30].

Studies in blood and liver from a rat model of adult obesity have shown that high fat diets resulted in increased body weight, glucose intolerance, overt hepatic steatosis, and increased hepatic oxidative stress with xanthine oxidase (XO) or NADPH oxidase-dependent ROS production in the onset of oxidative stress-dependent obesity, glucose intolerance, and hepatic steatosis process [80]. In humans, women with obesity have demonstrated reduced erythrocyte antioxidant enzyme activities, including superoxide dismutase, catalase and glutathione peroxidase, together with increased plasma lipid peroxidation [81]. Conversely, other studies have reported increased catalase activity and a trend toward elevated glutathione peroxidase activity [82], reflecting, similar to our findings in *fa/fa* rats, the heterogeneity and complexity of redox alterations associated with obesity.

### 4.4. Zucker Lean (fa/+) and Wistar Control Rats

Employing both *Zucker lean* rats and *Wistar* rats as control groups allowed us to identify that, for certain immune parameters, such as natural killer cell activity, lean rats more closely resembled obese *fa/fa* rats. *Zucker lean* rats also showed reduced lymphocyte proliferation similar to obese animals when compared with *Wistar* rats, a pattern that extended to redox parameters, particularly in the spleen.

Although *Zucker lean* rats (*fa/+*) share the same genetic background as (*fa/fa*) rats, they do not represent a fully normal physiological control, since they exhibit subclinical metabolic alterations derived from carrying the heterozygous mutation in the leptin receptor. Several studies have shown that (*fa/+*) rats may present differences in body weight, energy metabolism, basal inflammation, and immune function compared with non-mutated rats, which limits their validity as a truly “healthy” physiological control [30].

Moreover, it was demonstrated that *Wistar* rats could serve as a more appropriate control for aged *Zucker Diabetic Fat [ZDF* (*fa/fa*)*]* rats than the commonly used *ZDF* (*fa/+*) rats, which showed an increase in left ventricular weight, carbonyl stress markers in the left myocardium and MMP2 activity in both ventricles, indicating heart remodelling processes compared with the *Wistar* control group [35].

In contrast, *Wistar* rats are widely recognized as a standard reference model in studies of immunology, metabolism, and aging, as they do not carry mutations affecting appetite regulation, leptin sensitivity, or energy homeostasis. Their inclusion makes it possible to distinguish which alterations observed in *fa/fa* rats are truly due to obesity and metabolic dysfunction, and which may simply be a consequence of the *Zucker* genetic background [83].

### 4.5. Zucker fa/fa Rats as a Suggesting Model of Premature Aging

Obesity involves excessive adipose tissue accumulation, which disrupts metabolic and endocrine functions and alters adipokine secretion, contributing to chronic low-grade inflammation [70]. Additionally, dysregulation of both innate and adaptive immunity in obesity further amplifies this inflammatory state, reflecting the close interplay between metabolism and immune function [84].

The present study demonstrates that obese *Zucker fa/fa* rats develop marked physiological abnormalities, including increased body weight and altered organ weights. They also exhibit impaired innate and adaptive immune responses and disturbances in redox state. The accelerated immunosenescence observed in adult *fa/fa* rats, together with the oxidative and inflammatory stress characteristic of obesity, reflects key features of biological aging [85]. Since the immune parameters assessed have been proposed as indicators of health status, aging rate, and predictors of longevity [85,86,87], these findings support the hypothesis that adult *Zucker fa/fa* rats could constitute a model of premature aging.

The parameters studied have been evaluated at a single age point preventing prompt a complete evaluation of premature ageing. However, we can still state that, given the existing research with these parameters in various rat and mouse models that confirm that they are markers of premature aging, the results show that premature aging seems to occur in *fa/fa* rats. These animals in adulthood have values for the analyzed parameters more typical of older animals, in comparison to the controls, which points to premature aging. In fact, in several rodent models of premature and accelerated aging, immune functions such as NK activity, lymphoproliferative response, phagocytosis, chemotaxis and IL-2 production, which decline with chronological aging, at adult age are lower than in the corresponding controls and more similar to those in old animals [85]. All of this similarly occurs with oxidative parameters. Thus, due to the numerous studies in which the analyzed parameters have been shown to be in adult rodents of different models of premature aging, with altered values compared to the controls, we suggest that adult *fa/fa* rats show characteristics of premature aging.

### 4.6. Novelty of the Current Study and Future Research Lines

To the best of our knowledge, studies in the *Zucker fa/fa* rat model have primarily focused on isolated aspects of obesity-associated dysfunction, focusing separately on immune alterations, inflammatory mediators, or oxidative stress markers independently. In many cases, these analyses have also been restricted to a single tissue (most commonly adipose tissue) or to selected immune cell populations. However, given the strong biological interconnection between these processes, such fragmented approaches may limit the understanding of the mechanisms underlying obesity.

In this context, our present study was specifically designed to provide a comprehensive and systemic evaluation of the complex interactions underlying obesity-associated immunosenescence. To this end, the present study provides a comprehensive and integrative evaluation by simultaneously assessing multiple innate and adaptive immune functions, cytokine profiles, and redox parameters across multiple immunologically and metabolically relevant organs. This multidimensional approach allows for a more physiologically relevant and integrative characterization of the interaction between immune dysfunction, inflammation, and oxidative stress in the *Zucker* (*fa/fa*) model, which are tightly interconnected processes in obesity and aging, and has not been extensively addressed in previous studies.

We believe that this integrative perspective represents a key strength of the study, as it allows the identification of systemic alterations and potential interactions between pathways that cannot be captured when these parameters are evaluated in isolation. This is particularly relevant in the context of obesity as a model of premature immunosenescence, a condition characterized by the convergence of immune impairment, chronic inflammation, and oxidative stress. Furthermore, given that this immunosenescence is involved in “oxi-inflamm-aging” and the rate of aging, its presence in adult *Zucker* (*fa/fa*) rats supports our proposal that these animals could exhibit premature aging.

In this context, further work is needed to strengthen this hypothesis. It would have been interesting to include animals of both genders, to determine possible differences in the responses. Similarly, the claim of premature aging, that we are suggesting with our results, must be confirmed by other senescence markers.

## 5. Conclusions

This research demonstrates that *Zucker* (*fa/fa*) rats constitute an experimental model for immunosenescence as a consequence of obesity and the metabolic disturbances associated with type 2 diabetes. Adult *Zucker* (*fa/fa*) rats exhibited marked alterations in immune function and redox homeostasis across multiple organs, including reduced phagocytic and natural killer activity, impaired lymphoproliferative responses, dysregulated cytokine secretion, and significant oxidative imbalance, suggesting that these animals could constitute a model of premature aging. These findings reinforce the close relationship between obesity, inflammation, and oxidative stress and illustrate how severe metabolic dysfunction can disrupt both innate and adaptive immunity. Altogether, this integrated immunological and redox characterization provides a valuable insight into the systemic impact of obesity in this model and offers a robust framework for future studies exploring the mechanisms connecting metabolic disease with immune dysregulation.

## Figures and Tables

**Table 1:** Absolute weight (g) and relative percentage of each organ (collected at sacrifice) in *Zucker lean* (*fa/+*), obese (*fa/fa*) and *Wistar* (WT) rats. Data are expressed as mean ± S.E. Statistical analysis was performed by one-way ANOVA followed by Tukey’s post hoc test for homogeneous variances or Tamhane’s T2 post hoc test for unequal variances. *** *p* < 0.001, * *p* < 0.05 compared to the corresponding values in *Zucker lean* rats. ●●● *p* < 0.001, ●● *p* < 0.01, ● *p* < 0.05 compared to the corresponding values in *Wistar* rats.

	Absolute Weight (g)			Relative Weight (%)		
Organ	*Wistar* (WT)	*Zucker Lean* (*fa/+*)	*Zucker* (*fa/fa*)	*Wistar* (WT)	*Zucker Lean* (*fa/+*)	*Zucker* (*fa/fa*)
White Adipose Tissue	18.82 ± 1.25	9.19 ± 0.41 ●●●	33.92 ± 1.59 ***/●●●	3.57 ± 0.22	1.99 ± 0.90 ●●●	4.69 ± 0.16 ***/●●
Liver	15.33 ± 0.35	13.35 ± 0.26 ●●	26.41 ± 1.33 ***/●●●	2.92 ± 0.07	2.88 ± 0.04	3.65 ± 0.13 ***/●●●
Spleen	0.91 ± 0.02	0.69 ± 0.03 ●●●	0.94 ± 0.04 ***	0.17 ± 0.004	0.15 ± 0.006 ●●	0.13 ± 0.005 */●●●
Thymus	0.76 ± 0.05	0.61 ± 0.03 ●	1.87 ± 0.08 ***/●●●	0.15 ± 0.01	0.13 ± 0.01	0.26 ± 0.01 ***/●●●

**Table 2:** Basal proliferation and mitogen-induced responses (cpm) to Con A (1, 5 μg/mL), LPS (3, 5 μg/mL), and PHA (25, 50 μg/mL) in spleen and thymus lymphocytes from *Zucker lean* (*fa/+*), obese (*fa/fa*) and *Wistar* (WT) rats. Data are expressed as mean ± S.E. Statistical analysis was performed by one-way ANOVA followed by Tukey’s post hoc test for homogeneous variances or Tamhane’s T2 post hoc test for unequal variances. ●● *p* < 0.01, ● *p* < 0.05 compared to the corresponding values in *Wistar* rats.

Lymphoproliferation (cpm)						
Mitogens	Spleen			Thymus		
	*Wistar* (WT)	*Zucker Lean* (*fa/+*)	*Zucker* (*fa/fa*)	*Wistar* (WT)	*Zucker Lean* (*fa/+*)	*Zucker* (*fa/fa*)
BASAL	3928 ± 375	3667 ± 478	3523 ± 457	3037 ± 357	3082 ± 391	3694 ± 574
Con A (1 µg/mL)	6447 ± 969	4230 ± 514	3670 ± 454 ●	5122 ± 570	3649 ± 555	4782 ± 878
Con A (5 µg/mL)	6679 ± 1126	3781 ± 475 ●	3690 ± 449 ●	6940 ± 739	2798 ± 287 ●●	3881 ± 725 ●
LPS (3 µg/mL)	4961 ± 793	3146 ± 275	2805 ± 254	3674 ± 450	2681 ± 221	2684 ± 270
LPS (5 µg/mL)	4181 ± 380	3221 ± 286	3162 ± 389	4095 ± 547	3427 ± 573	3007 ± 250
PHA (25 µg/mL)	4946 ± 786	3375 ± 338	3259 ± 320	3177 ± 188	3817 ± 607	2763 ± 309
PHA (50 µg/mL)	4773 ± 900	3037 ± 439	3575 ± 481	3731 ± 203	3380 ± 436	2887 ± 303

**Table 3:** Total antioxidant capacity (U/mg tissue), total glutathione (nmol/mg tissue), glutathione peroxidase activity (mU/mg tissue), glutathione reductase activity (mU/mg tissue), and xanthine oxidase activity (mU/mg tissue) in spleen homogenates of *Zucker lean* (*fa/+*), obese (*fa/fa*) and *Wistar* (WT) rats. Data are expressed as mean ± S.E. Statistical analysis was performed by one-way ANOVA followed by Tukey’s post hoc test for homogeneous variances or Tamhane’s T2 post hoc test for unequal variances. ** *p* < 0.01 compared to corresponding values in *Zucker lean* rats. ●●● *p* < 0.001, ●● *p* < 0.01, ● *p* < 0.05 compared to corresponding values in *Wistar* rats.

Spleen	*Wistar* (WT)	*Zucker Lean* (*fa/+*)	*Zucker* (*fa/fa*)
Total Antioxidant Capacity (U/mg tissue)	0.20 ± 0.01	0.16 ± 0.01 ●	0.14 ± 0.01 ●●●
Total Glutathione (GSH) (nmol/mg tissue)	1.08 ± 0.03	1.12 ± 0.04	1.17 ± 0.04
Glutathione Peroxidase Activity (GPx) (mU/mg tissue)	295 ± 15	331 ± 19	360 ± 22 ●
Glutathione Reductase Activity (GR) (mU/mg tissue)	67 ± 3	71 ± 3	70 ± 3
Xanthine Oxidase Activity (XO) (mU/mg tissue)	6.94 ± 0.48	4.69 ± 0.19 ●●	6.09 ± 0.23 **

**Table 4:** Total antioxidant capacity (U/mg tissue), total glutathione (nmol/mg tissue), glutathione peroxidase activity (mU/mg tissue), glutathione reductase activity (mU/mg tissue) and xanthine oxidase activity (mU/mg tissue) in liver homogenates of *Zucker lean* (*fa/+*), obese (*fa/fa*) and *Wistar* (WT) rats. Data are expressed as mean ± S.E. Statistical analysis was performed by one-way ANOVA followed by Tukey’s post hoc test for homogeneous variances or Tamhane’s T2 post hoc test for unequal variances. ** *p* < 0.01, * *p* < 0.05 compared to corresponding values in *Zucker lean* rats. ●● *p* < 0.01, ● *p* < 0.05 compared to corresponding values in *Wistar* rats.

Liver	*Wistar* (WT)	*Zucker Lean* (*fa/+*)	*Zucker* (*fa/fa*)
Total Antioxidant Capacity (U/mg tissue)	0.28 ± 0.03	0.19 ± 0.01	0.22 ± 0.01
Total Glutathione (GSH) (nmol/mg tissue)	2.41 ± 0.09	2.72 ± 0.13	2.35 ± 0.06 *
Glutathione Peroxidase Activity (GPx) (mU/mg tissue)	1006 ± 39	1052 ± 63	818 ± 17 **/●●
Glutathione Reductase Activity (GR) (mU/mg tissue)	89 ± 4	88 ± 5	72 ± 3 */●●
Xanthine Oxidase Activity (XO) (mU/mg tissue)	4.25 ± 0.31	2.92 ± 0.27 ●	3.51 ± 0.13

## Data Availability

The original contributions presented in this study are included in the article. Further inquiries can be directed to the corresponding author.

## References

[1] Ambroselli D., Masciulli F., Romano E., Catanzaro G., Besharat Z.M., Massari M.C., Ferretti E., Migliaccio S., Izzo L., Ritieni A. (2023). New Advances in Metabolic Syndrome, from Prevention to Treatment: The Role of Diet and Food. Nutrients.

[2] Kassi E., Pervanidou P., Kaltsas G., Chrousos G. (2011). Metabolic syndrome: Definitions and controversies. BMC Med..

[3] Lemieux I., Després J.P. (2020). Metabolic Syndrome: Past, Present and Future. Nutrients.

[4] Galicia-Garcia U., Benito-Vicente A., Jebari S., Larrea-Sebal A., Siddiqi H., Uribe K.B., Ostolaza H., Martín C. (2020). Pathophysiology of Type 2 Diabetes Mellitus. Int. J. Mol. Sci..

[5] Chandrasekaran P., Weiskirchen R. (2024). The Role of Obesity in Type 2 Diabetes Mellitus-An Overview. Int. J. Mol. Sci..

[6] Ampofo A.G., Boateng E.B. (2020). Beyond 2020: Modelling obesity and diabetes prevalence. Diabetes Res. Clin. Pract..

[7] Villareal D.T. (2023). Editorial: Obesity and Accelerated Aging. J. Nutr. Health Aging.

[8] Palmer A.K., Jensen M.D. (2022). Metabolic changes in aging humans: Current evidence and therapeutic strategies. J. Clin. Investig..

[9] Ragusa F.S., Tanaka T., Veronese N., Mansueto P., Dominguez L.J., Barbagallo M., Ferrucci L. (2025). Weight of time: Exploring the link between obesity and aging. Aging Clin. Exp. Res..

[10] Subošić B., Zdravković V., Ješić M., Munjas J., Kovačević S., Guzonjić A., Mitrović J., Saso L., Đuričić I., Kotur-Stevuljević J. (2024). Childhood obesity accelerates biological ageing: Is oxidative stress a link?. Aging Clin. Exp. Res..

[11] Palmer A.K., Gustafson B., Kirkland J.L., Smith U. (2019). Cellular senescence: At the nexus between ageing and diabetes. Diabetologia.

[12] Cannizzo E.S., Clement C.C., Sahu R., Follo C., Santambrogio L. (2011). Oxidative stress, inflamm-aging and immunosenescence. J. Proteom..

[13] Kim N.H., Sim S.J., Han H.G., Yoon J.H., Han Y.H. (2025). Immunosenescence and age related immune cells: Causes of age related diseases. Arch. Pharm. Res..

[14] Yuliyanasari N., Rejeki P.S., Hidayati H.B., Subsomwong P., Miftahussurur M. (2024). The effect of intermittent fasting on preventing obesity-related early aging from a molecular and cellular perspective. J. Med. Life.

[15] Li Y., Tian X., Luo J., Bao T., Wang S., Wu X. (2024). Molecular mechanisms of aging and anti aging strategies. Cell Commun. Signal.

[16] Hunsche C., Hernandez O., De la Fuente M. (2016). Impaired Immune Response in Old Mice Suffering from Obesity and Premature Immunosenescence in Adulthood. J. Gerontol. A Biol. Sci. Med. Sci..

[17] Shahabi Nejad S., Zand H., Rastgoo S., Bahreini Boroujeni L.Z., Abedini Najafabadi M., Asadi S., Hamishe Bahar R., Shimi G. (2025). Obese plasma transfer accelerates cellular aging in the *C57BL/6* mouse model. Immun. Ageing.

[18] Ding C., Yimiti D., Sanada Y., Matsubara Y., Nakasa T., Matsubara K., Adachi N., Miyaki S. (2024). High fat diet induced obesity accelerates the progression of spontaneous osteoarthritis in senescence accelerated *mouse prone 8*. Mod. Rheumatol..

[19] Navarro M.D.C., Gálvez I., Hinchado M.D., Otero E., Torres-Piles S., Francisco-Morcillo J., de La Fuente M., Martín-Cordero L., Ortega E. (2024). Immunoneuroendocrine, stress, metabolic, and behavioural responses in high-fat diet induced obesity. Nutrients.

[20] Brunelli D.T., Boldrini V.O., Bonfante I.L.P., Duft R.G., Mateus K., Costa L., Chacon Mikahil M.P.T., Teixeira A.M., Farias A.S., Cavaglieri C.R. (2022). Obesity increases gene expression of markers associated with immunosenescence in obese middle aged individuals. Front. Immunol..

[21] Bryda E.C. (2011). The Mighty Mouse: The impact of rodents on advances in biomedical research. Mo. Med..

[22] Doulberis M., Papaefthymiou A., Polyzos S.A., Katsinelos P., Grigoriadis N., Srivastava D.S., Kountouras J. (2020). Rodent models of obesity. Minerva Endocrinol..

[23] Otani K., Funada H., Teranishi R., Okada M., Yamawaki H. (2022). Cardiovascular characteristics of *Zucker fatty diabetes mellitus* rats, an animal model for obesity and type 2 diabetes. Int. J. Mol. Sci..

[24] Yokoi N., Hoshino M., Hidaka S., Yoshida E., Beppu M., Hoshikawa R., Sudo K., Kawada A., Takagi S., Seino S. (2013). A novel rat model of type 2 diabetes: The *Zucker fatty diabetes mellitus ZFDM* rat. J. Diabetes Res..

[25] Tomassoni D., Martinelli I., Moruzzi M., Micioni Di Bonaventura M.V., Cifani C., Amenta F., Tayebati S.K. (2020). Obesity and age-related changes in the brain of the *Zucker Leprfa/fa* rats. Nutrients.

[26] De Castro N.M., Yaqoob P., de la Fuente M., Baeza I., Claus S.P. (2013). Premature impairment of methylation pathway and cardiac metabolic dysfunction in *fa/fa* obese *Zucker* rats. J. Proteome Res..

[27] Hwang I.K., Choi J.H., Nam S.M., Park O.K., Yoo D.Y., Kim W., Yi S.S., Won M.H., Seong J.K., Yoon Y.S. (2014). Activation of microglia and induction of pro-inflammatory cytokines in the hippocampus of type 2 diabetic rats. Neurol. Res..

[28] Raza H., John A., Howarth F.C. (2015). Increased oxidative stress and mitochondrial dysfunction in *Zucker diabetic* rat liver and brain. Cell Physiol. Biochem..

[29] Vrbjar N., Jasenovec T., Kollarova M., Snurikova D., Chomova M., Radosinska D., Shawkatova I., Tothova L., Radosinska J. (2022). Na,K-ATPase kinetics and oxidative stress in kidneys of *Zucker diabetic fatty* (*fa/fa*) rats depending on the diabetes severity-comparison with *lean* (*fa/+*) and *Wistar* rats. Biology.

[30] Radosinska D., Gaal Kovalcikova A., Gardlik R., Chomova M., Snurikova D., Radosinska J., Vrbjar N. (2024). Oxidative stress markers and Na,K-ATPase enzyme kinetics are altered in the cerebellum of *Zucker diabetic fatty fa/fa rats*: A comparison with *lean fa/+* and *Wistar* rats. Biology.

[31] Hong L., Zahradka P., Taylor C.G. (2024). Differential modulation by eicosapentaenoic acid (EPA) and docosahexaenoic acid (DHA) of mesenteric fat and macrophages and T cells in adipose tissue of obese *fa/fa Zucker* rats. Nutrients.

[32] Kruczkowska W., Gałęziewska J., Kciuk M., Gielecińska A., Płuciennik E., Pasieka Z., Zhao L.Y., Yu Y.J., Kołat D., Kałuzińska-Kołat Ż. (2024). Senescent adipocytes and type 2 diabetes-current knowledge and perspective concepts. Biomol. Concepts.

[33] Ruth M.R., Taylor C.G., Zahradka P., Field C.J. (2008). Abnormal immune responses in *fa/fa Zucker* rats and effects of feeding conjugated linoleic acid. Obesity.

[34] Tanaka S., Isoda F., Yamakawa T., Ishihara M., Sekihara H. (1998). T lymphopenia in genetically obese rats. Clin. Immunol. Immunopathol..

[35] Kollarova M., Chomova M., Radosinska D., Tothova L., Shawkatova I., Radosinska J. (2022). *ZDF* (*fa/fa*) rats show increasing heterogeneity in main parameters during ageing, as confirmed by biometrics, oxidative stress markers and MMP activity. Exp. Physiol..

[36] Dunn Z.S., Li Y.R., Yu Y., Lee D., Gibbons A., Kim J.J., Zhou T.Y., Li M., Nguyen M., Cen X. (2022). Minimally invasive preclinical monitoring of the peritoneal cavity tumor microenvironment. Cancers.

[37] Guayerbas N., Catalán M., Víctor V.M., Miquel J., De la Fuente M. (2002). Relation of behaviour and macrophage function to life span in a murine model of premature immunosenescence. Behav. Brain Res..

[38] De la Fuente M., Hernanz A., Guayerbas N., Alvarez P., Alvarado C. (2004). Changes with age in peritoneal macrophage functions. Implication of leukocytes in the oxidative stress of senescence. Cell Mol. Biol..

[39] De la Fuente M., Joyera N., Félix J., Díaz-Del Cerro E., Linillos-Pradillo B., Rancan L., Tresguerres J.A.F. (2024). Cannabidiol, a strategy in aging to improve redox state and immunity in male rats. Int. J. Mol. Sci..

[40] Tietze F. (1969). Enzymic method for quantitative determination of nanogram amounts of total and oxidized glutathione: Applications to mammalian blood and other tissues. Anal. Biochem..

[41] Arranz L., De Castro N.M., Baeza I., Giménez-Llort L., De la Fuente M. (2011). Effect of environmental enrichment on the immunoendocrine aging of male and female triple-transgenic *3xTg-AD* mice for Alzheimer’s disease. J. Alzheimers Dis..

[42] Lawrence R.A., Burk R.F. (1976). Glutathione peroxidase activity in selenium-deficient rat liver. Biochem. Biophys. Res. Commun..

[43] Massey V., Williams C.H. (1965). On the reaction mechanism of yeast glutathione reductase. J. Biol. Chem..

[44] Vida C., Rodríguez-Terés S., Heras V., Corpas I., De la Fuente M., González E. (2011). The aged-related increase in xanthine oxidase expression and activity in several tissues from mice is not shown in long-lived animals. Biogerontology.

[45] Løhr M., Folkmann J.K., Sheykhzade M., Jensen L.J., Kermanizadeh A., Loft S., Møller P. (2015). Hepatic oxidative stress, genotoxicity and vascular dysfunction in lean or obese *Zucker* rats. PLoS ONE.

[46] Hakkak R., Korourian S., Foley S.L., Erickson B.D. (2017). Assessment of gut microbiota populations in lean and obese *Zucker* rats. PLoS ONE.

[47] Marschall M.J.M., Ringseis R., Gessner D.K., Grundmann S.M., Most E., Wen G., Maheshwari G., Zorn H., Eder K. (2021). Effect of ecdysterone on the hepatic transcriptome and lipid metabolism in lean and obese *Zucker* rats. Int. J. Mol. Sci..

[48] Horwitz A., Birk R. (2023). Adipose tissue hyperplasia and hypertrophy in common and syndromic obesity-The case of BBS obesity. Nutrients.

[49] Durham H.A., Truett G.E. (2006). Development of insulin resistance and hyperphagia in *Zucker fatty* rats. Am. J. Physiol. Regul. Integr. Comp. Physiol..

[50] Nguyen T.T., Corvera S. (2024). Adipose tissue as a linchpin of organismal ageing. Nat. Metab..

[51] Kato Y., Sakoh M., Nagai T., Yoshida A., Ishida H., Inoue N., Yanagita T., Nagao K. (2022). Ozonated olive oil alleviates hepatic steatosis in obese *Zucker* (*fa/fa*) rats. J. Oleo Sci..

[52] Lopez Yus M., Hörndler C., Borlan S., Bernal Monterde V., Arbones Mainar J.M. (2024). Unraveling adipose tissue dysfunction: Molecular mechanisms, novel biomarkers, and therapeutic targets for liver fat deposition. Cells.

[53] Mousa M.F.M., Naeem M., Bibi S., Bülow R., Bahls M., Siewert Markus U., Töpfer P., Aghdassi A., Khattak M.N.K., Völzke H. (2024). Central obesity and fat-free mass are associated with a larger spleen volume in the general population. Ups. J. Med. Sci..

[54] Altunkaynak B.Z., Ozbek E., Altunkaynak M.E. (2007). A stereological and histological analysis of spleen on obese female rats fed with high-fat diet. Saudi Med. J..

[55] O’Shea D., Cawood T.J., O’Farrelly C., Lynch L. (2010). Natural killer cells in obesity: Impaired function and increased susceptibility to the effects of cigarette smoke. PLoS ONE.

[56] Pugliese G., Liccardi A., Graziadio C., Barrea L., Muscogiuri G., Colao A. (2022). Obesity and infectious diseases: Pathophysiology and epidemiology of a double pandemic condition. Int. J. Obes. Lond..

[57] Fei Q., Huang J., He Y., Zhang Y., Zhang X., Wang J., Fu Q. (2025). Immunometabolic interactions in obesity: Implications for therapeutic strategies. Biomedicines.

[58] Piening A., Ebert E., Gottlieb C., Khojandi N., Kuehm L.M., Hoft S.G., Pyles K.D., McCommis K.S., DiPaolo R.J., Ferris S.T. (2024). Obesity-related T cell dysfunction impairs immunosurveillance and increases cancer risk. Nat. Commun..

[59] Gulden G., Sert B., Teymur T., Ay Y., Tiryaki N.N., Mishra A.K., Ovali E., Tarhan N., Tastan C. (2023). CAR T cells with phytohemagglutinin (PHA) provide anti cancer capacity with better proliferation, rejuvenated effector memory, and reduced exhausted T cell frequencies. Vaccines Basel.

[60] Huldani H., Rashid A.I., Turaev K.N., Opulencia M.J.C., Abdelbasset W.K., Bokov D.O., Mustafa Y.F., Al-Gazally M.E., Hammid A.T., Kadhim M.M. (2022). Concanavalin A as a promising lectin-based anti-cancer agent: The molecular mechanisms and therapeutic potential. Cell Commun. Signal.

[61] Moriguchi S., Kato M., Sakai K., Yamamoto S., Shimizu E. (1998). Decreased mitogen response of splenic lymphocytes in obese *Zucker* rats is associated with the decreased expression of glucose transporter 1 (GLUT 1). Am. J. Clin. Nutr..

[62] Lamas O., Martinez J.A., Marti A. (2002). T helper lymphopenia and decreased mitogenic response in cafeteria diet induced obese rats. Nutr. Res..

[63] Lamas O., Martínez J.A., Marti A. (2003). Effects of a beta3 adrenergic agonist on the immune response in diet induced (cafeteria) obese animals. J. Physiol. Biochem..

[64] Tanaka S., Inoue S., Isoda F., Waseda M., Ishihara M., Yamakawa T., Sugiyama A., Takamura Y., Okuda K. (1993). Impaired immunity in obesity: Suppressed but reversible lymphocyte responsiveness. Int. J. Obes. Relat. Metab. Disord..

[65] Nieman D.C., Henson D.A., Nehlsen Cannarella S.L., Ekkens M., Utter A.C., Butterworth D.E., Fagoaga O.R. (1999). Influence of obesity on immune function. J. Am. Diet. Assoc..

[66] Gotoh K., Inoue M., Masaki T., Chiba S., Shimasaki T., Ando H., Fujiwara K., Katsuragi I., Kakuma T., Seike M. (2012). A novel anti inflammatory role for spleen derived interleukin 10 in obesity induced hypothalamic inflammation. J. Neurochem..

[67] Oleinika K., Slisere B., Catalán D., Rosser E.C. (2022). B cell contribution to immunometabolic dysfunction and impaired immune responses in obesity. Clin. Exp. Immunol..

[68] Valentine Y., Nikolajczyk B.S. (2024). T cells in obesity associated inflammation: The devil is in the details. Immunol. Rev..

[69] Grosso G., Laudisio D., Frias Toral E., Barrea L., Muscogiuri G., Savastano S., Colao A. (2022). Anti inflammatory nutrients and obesity associated metabolic inflammation: State of the art and future direction. Nutrients.

[70] Kim J.W., Kim J.H., Lee Y.J. (2024). The role of adipokines in tumor progression and its association with obesity. Biomedicines.

[71] Narmuratova G., Mukhalyiev Y., Deeney J.T., Narmuratova M., Abdolla N. (2025). Immune response in obesity and type 2 diabetes. J. Clin. Med. Kaz..

[72] Widjaja A.A., Lim W.W., Viswanathan S., Chothani S., Corden B., Dasan C.M., Goh J.W.T., Lim R., Singh B.K., Tan J. (2024). Inhibition of IL 11 signalling extends mammalian healthspan and lifespan. Nature.

[73] Barbé Tuana F., Funchal G., Schmitz C.R.R., Maurmann R.M., Bauer M.E. (2020). The interplay between immunosenescence and age related diseases. Semin. Immunopathol..

[74] Naomi R., Teoh S.H., Embong H., Balan S.S., Othman F., Bahari H., Yazid M.D. (2023). The role of oxidative stress and inflammation in obesity and its impact on cognitive impairments: A narrative review. Antioxid. Basel.

[75] Franceschi C., Bonafè M., Valensin S., Olivieri F., De Luca M., Ottaviani E., De Benedictis G. (2000). Inflamm aging: An evolutionary perspective on immunosenescence. Ann. N. Y Acad. Sci..

[76] Morita Y., Senokuchi T., Yamada S., Wada T., Furusho T., Matsumura T., Ishii N., Nishida S., Nishida S., Motoshima H. (2020). Impact of tissue macrophage proliferation on peripheral and systemic insulin resistance in obese mice with diabetes. BMJ Open Diabetes Res. Care.

[77] Deneke S.M., Fanburg B.L. (1989). Regulation of cellular glutathione. Am. J. Physiol..

[78] Lu S.C. (1999). Regulation of hepatic glutathione synthesis: Current concepts and controversies. FASEB J..

[79] Gasmi A., Nasreen A., Lenchyk L., Lysiuk R., Peana M., Shapovalova N., Piscopo S., Komisarenko M., Shanaida M., Smetanina K. (2024). An update on glutathione’s biosynthesis, metabolism, functions, and medicinal purposes. Curr. Med. Chem..

[80] Feillet Coudray C., Fouret G., Ebabe Elle R., Rieusset J., Bonafos B., Chabi B., Crouzier D., Zarkovic K., Zarkovic N., Ramos J. (2014). The mitochondrial targeted antioxidant MitoQ ameliorates metabolic syndrome features in obesogenic diet fed rats better than Apocynin or Allopurinol. Free Radic. Res..

[81] Amirkhizi F., Siassi F., Minaie S., Djalali M., Rahimi A., Chamari M. (2007). Is obesity associated with increased plasma lipid peroxidation and oxidative stress in women?. ARYA Atheroscler. J..

[82] Adenan D.M., Jaafar Z., Jayapalan J.J., Abdul Aziz A. (2020). Plasma antioxidants and oxidative stress status in obese women: Correlation with cardiopulmonary response. PeerJ.

[83] Patel S., Patel S., Kotadiya A., Patel S., Shrimali B., Joshi N., Patel T., Trivedi H., Patel J., Joharapurkar A. (2024). Age related changes in hematological and biochemical profiles of *Wistar* rats. Lab. Anim. Res..

[84] Shaikh S.R., Beck M.A., Alwarawrah Y., MacIver N.J. (2024). Emerging mechanisms of obesity associated immune dysfunction. Nat. Rev. Endocrinol..

[85] De la Fuente M., Miquel J. (2009). An update of the oxidation inflammation theory of aging: The involvement of the immune system in oxi inflamm aging. Curr. Pharm. Des..

[86] Ledón N., Añé-Kourí A.L., Ramos M.B., Lorenzo-Luaces P., Silva A., Pereira K., Lage A., Saavedra D. (2023). Immunosenescence and inflammatory markers in Cuban centenarians: Implications for survival. Aging Clin. Exp. Res..

[87] Singh A., Schurman S.H., Bektas A., Kaileh M., Roy R., Wilson D.M., Sen R., Ferrucci L. (2024). Aging and inflammation. Cold Spring Harb. Perspect. Med..

